# Esophageal Carcinoma Histology Affects Perioperative Morbidity Following Open Esophagogastrectomy

**DOI:** 10.1155/2008/389394

**Published:** 2009-02-05

**Authors:** Charles E. Woodall, Ryan Duvall, Charles R. Scoggins, Kelly M. McMasters, Robert C. G. Martin

**Affiliations:** Division of Surgical Oncology, Department of Surgery, James Graham Brown Cancer Center, University of Louisville School of Medicine, Louisville, KY 40292, USA

## Abstract

*Background*. Esophagectomy for esophageal cancer is being practiced routinely with favorable results at many centers. We sought to determine if tumor histology is a powerful surrogate marker for perioperative morbidity. *Methods*. Seventy three consecutive patients managed operatively were reviewed from our prospectively maintained database. *Results*. Adenocarcinoma (AC) was present in 52 (71%) and squamous cell (SCC) in 21 (29%). The use of neoadjuvant therapy was similar for the AC (34.62%) and SCC (42.86%) groups. The SCC group had a higher incidence of prior pulmonary disease than the AC group (23.8% versus 5.8%, resp.; *P* = .03). SCC patients were more likely to have a prolonged ICU stay than AC patients (*P* = .004) despite similar complication rates, EBL, and prognostic nutritional index. The SCC group did, however, experience higher grades of complications (*P* = .0053). *Conclusions*. Presence of SCC was the single best predictor of prolonged ICU stay and more severe complications as defined by this study. Only a past history of pulmonary disease was different between the two histologic subgroups.

## 1. Introduction

Esophagectomy for esophageal cancer is being practiced routinely with favorable results at many centers. Improvements in surgical technique and perioperative care have permitted a procedure once associated with high mortality rates to now be practiced with a low risk of postoperative death. However, studies continue to report high morbidity and there is now a focused effort to identify factors that may predict perioperative outcome.

Pulmonary complications are a major contributor to mortality in esophageal cancer and efforts to improve pulmonary hygiene have contributed to reduced perioperative mortality [1]. As an adjunct to further decrease these complications, the necessity of a thoracic incision when performing esophagectomy has long been debated. Proponents have often argued that its use enables a more complete lymphadenectomy, while opponents feel it contributes significantly to perioperative morbidity but this has not been shown to affect long-term prognosis [2].

Given the importance of pulmonary status on outcome, the ability to predict those patients at higher risk for pulmonary complications might then be of some benefit, not only in predicting their potential for morbidity, but also in choosing the approach that may mitigate these risks. Many feel that a minimally invasive approach may be the answer. Recently, investigators have reported promising results with minimally invasive approaches [3, 4]. While there is little debate regarding the role of surgical technique and comorbid conditions on the development of postoperative complications, little is known about the impact of tumor biology on morbidity. Based on recent a report suggesting that the esophageal histology may impact perioperative outcome [5], we sought to further delineate this relationship. This study is an attempt to recognize that tumor histology alone can identify patients more likely to suffer a complication and perhaps guide perioperative decision making.

## 2. Methods

The records of patients included in a prospectively maintained upper gastrointestinal malignancy database were reviewed for this institutional review board approved study. All patients undergoing esophagogastrectomy for esophageal carcinoma were included in the study, and all patients underwent a standard combined thoracic and abdominal esophagogastrectomy (Ivor-Lewis type) procedure. This review was performed under an IRB approved protocol from the University of Louisville Human Subjects Protection Office. All patients had undergone complete preoperative evaluation with CT chest/abdomen/ pelvis, endoscopic ultrasound, and in some, PET scanning. Most patients with preoperative T2 or greater, or N1 (by imaging) were given neoadjuvant chemoradiation with either 5-fluorouracil or combined 5 fluorouracil with cisplatin depending on the histology of the disease and standard radiation therapy dosing of 5040 cGy. Operative techniques were consistent across this study with the esophagogastrectomy performed through an abdominal incision first, with mobilization, celiac and supraceliac lymphadenectomy, pyloroplasty, and gastric conduit formation followed by a thoracic incision, with mobilization, thoracic lymphadenectomy, tumor resection, and thoracic anastomosis.

Variables evaluated included demographics (age, race, and gender), smoking history, alcohol history, histology, cancer staging, grade and type of complications, nutritional status, length of intensive care unit stay, and operative factors including estimated blood loss. Comorbidities, such as prior cardiac and pulmonary disease as well as history of tobacco and alcohol abuse as reported by the patient were also recorded. The prognostic nutritional index (PNI) advocated by Onodera et al. [6] was calculated to investigate the preoperative nutritional condition of the patients in both groups. It is calculated from the formula (giving a percentage) (10 × Albumin) + (0.005 × absolute lymphocyte count). Prior cardiac history was defined as any patient with a history of angina, previous coronary artery disease defined by cardiac catheterization, previous myocardial infarction, cardiac valve dysfunction requiring medication, or a history of congestive heart failure or tachyarrhythmia. Prior pulmonary disease history was defined as any patient with abnormal pulmonary function tests, history of asthma requiring daily meter dosed inhalers, or tobacco use greater than a 25-pack year history.

All postoperative complications and the length of hospital stay were prospectively entered into the database. Complications were identified prospectively and assigned a grade from 1 to 5 based on an established scale [7]. Examples of the grading of complications includes (1) uncomplicated urinary tract infection; (3) small, contained anastomotic leak requiring no further operative therapy or drainage procedures; (5) death. In instances where the grading was unclear, a score was assigned after review of the records and discussion between two of the senior authors. All in hospital and 90-day postoperative complications were evaluated with the most severe complication level recorded. Infectious complications were defined by a positive fluid (sputum, wound, urine, etc.) culture, with some criteria of a systemic inflammatory response (i.e., tachycardia, fever, and hypoxia)

Statistical analysis was performed with JMP software (SAS Institute, Cary, NC, USA). Analysis of variance, log-rank analysis, and Pearson correlation coefficient were used to determine significance, and a *P*-value <.05 was considered significant in this study.

## 3. Results

Seventy three consecutive patients undergoing combined abdominal and thoracic esophagogastrectomy for cancer were identified and included in the study, with a median age was 61 (range 26 to 80). 55 (75.3%) were male and 18 female (24.7%). Fifty four patients (74%) were Caucasian, 12 (16%) were African-American, and the race of 7 (9%) was not recorded. There were 3 (4.1%) perioperative deaths, all occurring in the AC group. Adenocarcinoma (AC) was present in 52 (71%) and squamous cell (SCC) in 21 (29%). In Caucasians, AC occurred more often than SCC (84% versus 47%, resp.; *P* = .004). Adenocarcinoma was also much more common in males (86%) than SCC (47%; *P* = .0007). The AC patients were slightly younger (59 versus 63) than those in the SCC group (*P* = .21) (Table 1).

Patients in the SCC group were significantly more likely to have a history of alcohol abuse (8/21, 38.1%) versus those in the AC group (6/52, 11.5%; *P* = .012). They were also more likely to have a history of pulmonary disease (asthma, COPD, pneumonia) than the AC group (23.8% versus 5.77%; *P* = .034). Interestingly, there was no difference in the rate of COPD between the two groups (2.8% versus 3.9%; *P* = .133) and no difference in rates of tobacco use (61.9% versus 69.2%; *P* = .5492), mean pack years (56.6% versus 51.0%; *P* = .573), or prior cardiac disease history (CAD, atrial fibrillation, prior MI, or percutaneous coronary intervention (PCI); 19.1% versus 25.1%; *P* = .5806). At the time of operation, median estimated blood loss was similar for both groups (551 mL for AC and 600 mL for SCC, *P* = .7626).

In the AC group, there were two (3.9%) patients with in situ disease, five (9.8%) with T0 disease, five (9.8%) T1s, 10 (19.6%) T2s, 27 (52.9%) with T3 disease, and 2 (3.9%) with T4 disease on final pathology (Table 2). The SCC group had a similar distribution: 2 (9.5%) T0s, 4 (19.0%) T1s, 2 (9.5%) T2s, 9 (42.8%) T3s, and 4 (19.1%) T4s; the differences were not significant (*P* = .2). Nodal staging for the AC group consisted of 24 patients (47%) with N0 disease, 25 (49%) with N1 disease, and 2 (3.9%) patients with N2 disease. In the SCC group, there were 14 (66.7%) with N0 disease and 7 (33.3%) with N1 disease. There were no patients with N2 disease in the SCC group. The differences were not statistically significant (*P* = .25). Metastatic disease was found in one patient in each group (*P* = .5).

The use of neoadjuvant chemoradiation between the AC and SCC groups was similar. Overall, 36% of patients received preoperative therapy: 18 (34%) of the AC group and 9 (42%) of the SCC group (*P* = .5). A similar proportion of patients in each group had experienced weight loss prior to undergoing operative therapy: 55.7% (29) of AC and 61.9% (13) of SCC. There was a trend in patients with AC to have a BMI greater than 20, while patients with SCC tended to have a BMI less than 20 (*P* = .056). The difference in mean BMI among groups was significant, however. In the AC group, the mean was 26.7 while in the SCC group it was 22.889 (*P* = .0299). Also, female patients tended to have a decreased BMI (81.2%) versus male patients (36.7%); this was significant (*P* = .002) as well. The African-American patients also had lower BMI (80% less than 20) than Caucasians (42.8% less than 20; *P* = .06).

There were 65 independently identified complications among 52 of the 73 patients comprising the cohort (Table 3). Complications were graded on the basis of an established scale. Seventy percent of patients experienced some sort of complication: 9 (12%) were grade 1, 12 (16%) were grade 2, 23 (31.5%) were grade 3, 1 (1%) was grade 4, and 6 (8%) were grade 5. The grade 5 complications included the three aforementioned deaths. The rate of complications between the AC and SCC groups (65.4% versus 85.7%) approached statistical significance (*P* = .0693). However, when the patients with no complications were excluded, the distribution of the most severe complication in each patient (grade 1 or 2 versus grade 3, 4, or 5) revealed a statistically significant disproportion with more severe complications occurring the SCC group versus the AC group (64.7% versus 55.9%, *P* = .0053). No difference existed among races or genders in complications. Pulmonary complications (including pneumonia) were the most predominant, comprising 29.3% of all complications. These were most strongly associated with prior cardiac disease (*P* = .056), and not with prior COPD history (*P* = .225), pulmonary history (*P* = .336), histologic subtype (*P* = .503), or increasing pack-year history of tobacco (*P* = .609). Esophageal leak (a grade 3 complication) was the second most common complication, with 10 (13.7%) occurrences. There was no significant difference in leak rate among histologic groups (*P* = .805) or anastomosis type (*P* = .965).

Among the patients in the SCC group, the median PNI was 40.95 (range 27.56 to 61.36); in the AC group the median was 39.78 (range 27.75 to 57.16; *P* = .6983). PNI did not appear to affect morbidity. In the group of patients with a PNI less than 40, there was a complication rate of 70.83%; in those with a PNI greater than 40, the rate was 79.17% (*P* = .5042). The distribution by grade of complications was equivalent between those patients with a PNI of greater than 40 versus those with a PNI less than 40 (*P* = .9986). Patients with a PNI less than 40 were also not any more likely to have a major (grade 3, 4, or 5) complication versus those with a PNI greater than 40 (*P* = .9396). The differences in distributions of pulmonary (*P* = .7452) and anastomotic leak (*P* = .1501) were also not statistically significant between the PNI groups.

Despite the similarities among the groups in total complications, SCC patients were more likely to have a 3-day or longer ICU stay than AC patients (*P* = .004). The higher incidence of pulmonary disease in these patients was the largest contributor to this finding (*P* = .0016). However, prior tobacco use (*P* = .8254), total pack years (*P* = .1286) or cardiac disease (*P* = .5803) were not associated with a prolonged ICU course. SCC was more likely in patients and 60 years of age (*P* = .004) but age was not an independent factor for prolonged ICU stay.

## 4. Discussion

Advances in technique and patient care have lead to overall decreases in esophagectomy mortality in the last 5 years [8]. However, morbidity remains high (60% in some series) and appears to be associated with tumor histology. Thus the aim of the present study was to delineate the role of tumor histology in regards to perioperative morbidity and possibly preoperative decision making. Our study suggests that tumor histology may be a significant predictor of morbidity, primarily as a surrogate for increased pulmonary complications. These findings are supported by a similar study from the United Kingdom [9], and might function as an adjunct to other prognostic scoring systems [10].

Despite advances in surgical technique and perioperative care, the types of complications in esophageal cancer are fairly consistent [11] (Table 4). Pulmonary morbidity and anastomotic leaks remain the most common [12]; both of which can significantly effect a patient's long-term quality of life [13] when they occur. Pulmonary complications contribute to most cases of mortality in esophageal cancer and active efforts to minimize their effects have been attributed as one of the most significant causes of decreased perioperative mortality [14]. The historically dreaded anastomotic leak has been delegated to a lesser standing; this is in large part due to new minimally invasive endoscopic techniques that have been described for the management of leaks [15], making what was once a devastating problem somewhat more easily managed and no longer a source of increased mortality or decreased long-term survival [16]. However, the pulmonary problems are more difficult in a population in which, at least in the case of SCC, patients are more likely to be smokers and thus more likely to carry a diagnosis of underlying COPD. This was likely the root cause of the prolonged intensive care unit stays seen in the SCC cohort in this study.

The perioperative risks for patients with COPD are well known [17]. However, age, operative duration, and proximal tumor location have also been identified as factors contributing to pulmonary morbidity [18], of which all are more likely to be associated with SCC histology than AC. Despite an efficient resection, patients who suffer complications are at increased risk of surgical oncotaxis [19], the acceleration of their disease caused by operative factors. The patients reported in this study by Hirai et al. had earlier metastasis and poorer long-term outcomes. Therefore, at least from one study, minimizing morbidity is important not only from a short-term perioperative perspective but also from a long-term cancer prognosis standpoint. Although it should be noted that another study, by Ferri et al., showed an increased short-term mortality in SCC patients suffering a complication, there are no long-term effects in those that survived [20].

Nutrition remains the focus in many studies of esophagectomy, but its role in morbidity is somewhat unclear. Few other malignancies affect the nutritional status of the patient prior to diagnosis more than esophageal cancer, and are thus a potential powerful marker of surgical outcome. Most surgeons would associate esophageal cancer with malnutrition, noting the diminution in the ability of the patient to take in adequate calories in addition to the wasting normally seen with other malignancies, and thus try to supplement feedings. Advocates of this approach stress the benefits of preoperative enteral supplementation. The perioperative advantages of this were identified in a paper by Nozoe demonstrating decreased complications and better long-term survival in patients with higher prognostic nutritional index (PNI), a mathematical computation of the patients albumin and absolute lymphocyte count [21]. As discussed by Onodera in the initial description of PNI, a minimum value of 40 is recommended prior to undertaking an esophageal resection. In the present study, a PNI of less than 40 did not adversely affect outcomes and led to no increase in morbidity.

At the opposing end of the spectrum, increasing BMI has been attributed to the increasing incidence of AC. Even significantly, overweight patients may be relatively catabolic and consideration for supplementation should be given in this population as well. Fortunately, increasing patient BMIs has not been associated with poorer operative or disease related outcomes. Also, in at least one large study of 400 patients, nutritional status as determined by BMI, PNI, weight loss, and other factors had no value in predicting perioperative complications [22].

Over time, multimodality treatment of esophageal cancer has improved, offering increased long-term survival [23]. Better results have been noted for factors most would identify as predictive of long-term success in any cancer, including low AJCC stage, R0 resection, and M0 status. Neoadjuvant therapy is gaining acceptance, as it can be given safely, is generally better tolerated than adjuvant therapy, and does not affect operative morbidity or mortality. SCC can be treated safe and effectively with multimodality therapy, providing durable results even for patients with positive nodal disease as well as those from Asian studies discovered to have early tumors. Regardless of the physician's opinion in regards to the timing of additional therapy, most agree that esophageal squamous and adenocarcinoma are not purely surgically treated diseases and that some form of multimodality treatment is needed to extend quality of life time. Therefore, from a surgical perspective, optimizing patient selection and operative technique are important so that patients may recover quickly and go on to their additional therapy.

Technical advances have allowed for refinement in the techniques in esophageal surgery to reduce perioperative morbidity and mortality [24]. What technology to apply on a case-to-case basis is a somewhat more difficult question to answer. Recent studies have also served to benchmark expected courses for patients with AC, and the outcomes for all esophageal resections has improved significantly [25]. Because of this, any changes in techniques or approach need to be critically reviewed. Choice of operative approach has been extensively studied, but until recently has focused on the transhiatal versus transthoracic approach [2] or technical factors such as the location of the conduit in the mediastinum. With the public's growing interest in minimally invasive approaches, coupled with new techniques and instrumentation, minimally invasive esophagectomy has been proven safe and feasible both in the United States and abroad [3]. It also does not adversely affect long-term survival, a question that has been repeatedly raised when laparoscopic approaches are used to address surgical oncology diagnoses. The difficulty is that there are a variety of techniques and combinations of approaches reported as “minimally invasive,” with no standardized definition and any real benefit over traditional techniques has yet to be proven. Most advocates of this approach perceive decreased pulmonary morbidities and improved pulmonary therapy as the main advantages, but some studies have questioned this benefit. Preoperative pulmonary evaluations have tended to focus on pulmonary factors alone, including smoking and COPD. However, given these factors in a high-risk subgroup (SCC), there might be more of an advantage for minimally invasive techniques and perhaps we should evaluate these patients for this approach.

The limitations of this study are the small sample size of the SCC patients. This limits the impact of the data presented, but does not limit the fact that these two types of histologies are both biologically different and physiologically different and treating physicians should be aware of these differences and the impact that these play on perioperative outcomes.

## 5. Conclusion

The presence of SCC was the single best predictor of prolonged intensive care unit stay and more severe complications as defined by this study. Only a past history of pulmonary disease was different between the two histologic subgroups. No other factor, including sex, gender, age, or nutritional status was predictive of this outcome. Esophageal histology should therefore be considered in the perioperative care of patients and may in the future be used to guide operative strategies.

## Figures and Tables

**Table 1:** Adenocarcinoma and squamous cell carcinoma groups patient demographics.

	Adenocarcinoma, *n* (%)	Squamous cell carcinoma, *n* (%)	*P*-value
*n*	52 (71%)	21 (29%)	
Median age	59	63	.21
Caucasian race	44 (8462%)	10 (47.62%)	**.0004**
Male gender	45 (86.54%)	10 (47.62%)	**.0007**
Alcohol abuse	6 (11.54%)	8 (38.1%)	**.012**
Tobacco use	36 (69.23%)	13 (61.9%)	.5492
Prior cardiac disease	13 (25%)	4 (19.05%)	.573
Prior pulmonary disease	3 (5.77%)	5 (23.81%)	**.034**
FEV1 <75%	3 (5.77%)	4 (19%)	.06

**Table 2:** Adenocarcinoma and squamous cell carcinoma tumor features and perioperative data.

		Adenocarcinoma, *n* (%)	Squamous cell carcinoma, *n* (%)	*P*-value
T stage				.2032

	T0	5 (9.80%)	2 (9.52%)	
	Tis	2 (3.92%)	0	
	T1	5 (9.80%)	4 (19.05%)	
	T2	10 (19.61%)	2 (9.52%)	
	T3	27 (52.94%)	9 (42.86%)	
	T4	2 (3.92%)	4 (19.05%)	

N stage				.1933

	N0	24 (47.06%)	14 (66.67%)	
	N1	25 (49.02%)	7 (33.33%)	
	N2	2 (3.92%)	0	

Neoadjuvant therapy		18 (34.62%)	9 (42.86%)	.5114
Weight loss		29 (55.77%)	13 (61.90%)	.6301
Mean BMI		26.72	22.89	**.0299**
Mean PNI		33.61	33.00	.6921
Epidural anesthesia		44 (85%)	18 (87%)	.78

Anastomosis				.8892

	Stapled	17 (33.33%)	6 (31.58%)	
	Sewn	34 (66.67%)	13 (68.42%)	
Mean EBL		551.065	600.000	.7626
Margin Pos		1 (2%)	1 (4%)	.08
Time from OP to extubation		0.5 (0–48)	1 (0–72)	.86
Complications		34 (65.38%)	18 (85.71%)	.0693

Grade of complications				**.0053**

	1 or 2	15 (44.12%)	6 (35.29%)	
	3, 4, or 5	19 (55.88%)	11 (64.71%)	
ICU stay >3 days		23 (47.92%)	16 (76.19%)	**.0259**

**Table 3:** All inhospital and 90-day postoperative complications and grade by histology.

Complication	Adeno	SCC
*n*	34/52 (65%)	17/21 (81%)

Grade 1:	**6**	**3**
* *Pneumonia	—	1
* *Fever	1	1
* *Partial cord paralysis	—	1
* *Anastomotic leak	1	—
* *Hypertension	1	—
* *Decubitus ulcer	1	—
* *Hypovolemia	1	—
* *Urinary tract infection	1	—

Grade 2:	**9**	**3**
* *Fever	1	1
* *Mediastinitis	—	1
* *Prolonged enteral feeding	—	1
* *Excessive pain	1	—
* *Pneumonia	2	—
* *Pleural effusion	1	—
* *Readmission	1	—
* *Anastomotic leak	1	—
* *Atrial fibrillation	2	—

Grade 3:	**15**	**8**
* *Pleural effusion, pneumonia	—	1
* *Anastomotic leak, EtOH withdrawal	—	1
* *Anastomotic leak, pleural effusion	—	1
* *Anastomotic leak	3	2
* *Delayed gastric emptying	—	1
* *Pleural effusion	1	1
* *Respiratory compromise	1	1
* *Confusion, esophageal leak	1	—
* *Confusion, pneumonia, respiratory failure	1	—
* *Pleural effusion, atelectasis	1	—
* *Anastomotic leak, pneumonia	1	—
* *Anastomotic leak, pneumonia, SVT	1	—
* *Anastomotic leak, evisceration	1	—
* *Anastomotic leak, paraesophageal hernia	1	—
* *SVT	1	—
* *Pneumonia	1	—
* *Hemorrhage	1	—

Grade 4:	**1**	**0**
* *Anastomotic leak	1	—

Grade 5:	**3**	**3**
* *Anastomotic leak	—	1
* *Pneumonia	—	1
* *Pulmonary embolus	1	—
* *Death	2	1

**Table 4:** Recent studies of morbidity in SCC patients undergoing esophagectomy.

Author	Year	*n*	SCC %	SCC approach	SCC pulmonary	SCC anastomotic leak	SCC median EBL (mL)	SCC ICU stay (days)	SCC mortality
Whooley	2001	710	100%	TTE (100%)	32%	3.5%	832	—	11%
Ferguson	2002	290	34.5%	—	39%	—	—	—	—
Fang	2003	441	>90%	3 Field (100%)	7.3%	32.65%	587.5–642.1	—	2.5%
Law	2004	421	100%	TTE (83%)	15.9%	3.1%	700	—	1.4%
Alexiou	2006	621	31.72%	TTE (55%)	18.3%	8.6%	—	—	8.1%
Woodall	2007	73	29%	TTE (100%)	28.57%	26.32%	600	6	0%
